# Effects of High-Definition Transcranial Direct-Current Stimulation on Resting-State Functional Connectivity in Patients With Disorders of Consciousness

**DOI:** 10.3389/fnhum.2020.560586

**Published:** 2020-09-23

**Authors:** Rui Zhang, Lipeng Zhang, Yongkun Guo, Li Shi, Jinfeng Gao, Xinjun Wang, Yuxia Hu

**Affiliations:** ^1^School of Electrical Engineering, Zhengzhou University, Zhengzhou, China; ^2^Henan Key Laboratory of Brain Science and Brain-Computer Interface Technology, Zhengzhou, China; ^3^The Fifth Affiliated Hospital of Zhengzhou University, Zhengzhou, China; ^4^Department of Neurosurgery, Zhengzhou Central Hospital Affiliated to Zhengzhou University, Zhengzhou, China; ^5^Department of Automation, Tsinghua University, Beijing, China; ^6^Beijing National Research Center for Information Science and Technology, Beijing, China

**Keywords:** disorders of consciousness, high-definition transcranial direct-current stimulation, electroencephalography, coma recovery scale-revised scores, resting-state brain network

## Abstract

Recently a positive treatment effect on disorders of consciousness (DOCs) with high-definition transcranial direct-current stimulation (HD-tDCS) has been reported; however, the neural modulation mechanisms of this treatment’s efficacy need further investigation. To better understand the processing of HD-tDCS interventions, a long-lasting HD-tDCS protocol was applied to 15 unresponsive wakefulness syndrome (UWS) patients and 20 minimally conscious states (MCS) patients in this study. Ten minutes of resting-state electroencephalograms (EEGs) were recorded from the patients, and the coma recovery scale-revised scores (CRS-Rs) were assessed for each patient from four time-points (T0, T1, T2, and T3). Brain networks were constructed by calculating the EEG spectral connectivity using the debiased weighted phase lag index (dwPLI) and then quantified the network information transmission efficiency by graph theory. We found that there was an increasing trend in local and global information processing of beta and gamma bands in resting-state functional brain networks during the 14 days of HD-tDCS modulation for MCS patients. Furthermore, the increased functional connectivity not only occurred in the local brain area surrounding the stimulation position but was also present across more global brain areas. Our results suggest that long-lasting HD-tDCS on the precuneus may facilitate information processing among neural populations in MCS patients.

## Introduction

Understanding the neural mechanisms of recovery processes in patients with chronic disorders of consciousness (DOCs) is a daunting challenge for modern neuroscience (Chennu et al., 2016). DOCs can be subdivided into the following based neurobehavioral assessments: comas, unresponsive wakefulness syndrome (UWS; Laureys et al., 2010), minimally conscious state (MCS; Giacino et al., 2002), and emergence of the minimally conscious state (EMCS). The MCS can be further divided into MCS minus (MCS−) and MCS plus (MCS+; Bruno et al., 2012). Previous studies have used both pharmacological and non-pharmacological interventions—such as median nerve electrical stimulation, transcranial magnetic stimulation, spinal cord stimulation, and deep brain stimulation—to treat DOCs, but few outcome measurements have been assessed in such studies (Schiff et al., 2007; Giacino et al., 2012; Della Pepa et al., 2013; Yamamoto et al., 2013; Cossu, 2014; Thibaut et al., 2014; Tucker and Sandhu, 2016).

Recently, due to the use of invasive stimulation techniques have the ethical and technical limitations, the transcranial direct-current stimulation (tDCS) has been widely investigated to improve the consciousness level of DOC patients (Zhang and Song, 2018). This technique not only could avoid surgical risks but also is lower costs for patients compared to surgical interventions (Huang et al., 2019). Specifically, with a weak current that flows through the cerebral cortex from the anode to the cathode, the cortical excitability at stimulation sites were modulated by tDCS. Anodal tDCS increases neuronal activation by sub-threshold neuronal membrane polarization, while cathodal tDCS decreases cortical excitability (Lefaucheur et al., 2017). Several previous studies have confirmed the positive effect of employing tDCS on DOCs’ clinical improvement (Bai et al., 2017; Huang et al., 2017; Zhao et al., 2017). Currently, the most commonly used stimulation target is the left DLPFC (Angelakis et al., 2014; Estraneo et al., 2017), and these inspiring results indicate tDCS seems promising for the rehabilitation of DOC patients. Besides, the posterior parietal cortex, cerebellar cortex, and precuneus have also been selected in some studies (Huang et al., 2017; Cai et al., 2019). However, it is still a long way before tDCS becoming a formal clinical application for treating DOCs.

High-definition tDCS (HD-tDCS) is a new kind of neuromodulation technique that—instead of using two large pad electrodes as in traditional tDCS—uses small and compact scalp electrodes. Compared with tDCS, HD-tDCS is more precise and results in focal neural modulation and specific behavioral changes (Dmochowski et al., 2011; Villamar et al., 2013; Shekhawat and Vanneste, 2018). HD-tDCS has been proved to be effective to improve working memory, verbal learning, motor function, and control of pain and tinnitus (Borckardt et al., 2012; Kuo et al., 2013; Donnell et al., 2015; Nikolin et al., 2015; Shekhawat et al., 2016). Although these previous results suggest beneficial effects of HD-tDCS, the neural mechanisms of HD-tDCS on DOC have remained unclear.

Since electroencephalograms (EEGs) are convenient, high-cost performance, and comparatively suitable to work at the patient’s bedside, they play an irreplaceable role in DOC studies. Additionally, graph theory has also been widely used in the field of neuroscience recently (Bullmore and Sporns, 2009; Bullmore and Bassett, 2011; Fornito et al., 2013). Based on these approaches, recent studies have shown that both qualitative assessments by experts (Forgacs et al., 2014; Estraneo et al., 2016; Piarulli et al., 2016; Bagnato et al., 2017) and quantitative assessments using quasi-automated machine learning (Sitt et al., 2014) could be valid in evaluating the state of consciousness of patients by EEGs. Furthermore, some studies have reported that different resting-state functional networks characterize healthy and DOC subjects and that their consciousness levels are relevant to their network properties (Chennu et al., 2014, 2017; Malagurski et al., 2019). These findings suggest that using resting-state EEGs and graph theory to study DOC patients is feasible and may help to further understand and treat DOCs.

In the current study, resting-state EEGs and coma recovery scale-revised scores (CRS-Rs) were applied for assessing the treatment efficacy of long-lasting HD-tDCS in patients with DOC. We aimed to verify the efficacies of HD-tDCS treatment *via* electrophysiological evidence. Additionally, we investigated whether brain areas closer to the stimulus site were more likely functional recovery.

## Materials and Methods

### Patients

Thirty-five hospitalized DOC patients (13 females and 22 males, 51.7 ± 14.1 years old) were included in this study (Table 1). These patients did not show cortical lesions in the precuneus. Data collection was completed in the Zhengzhou Central Hospital Affiliated to Zhengzhou University. With the CRS-Rs, the score of the patient’s consciousness was assessed (Giacino et al., 2004) and all CRS-R were performed by trained physicians at T0, T1, T2, and T3. Each patient was evaluated by the same physician. In this study, we divided the MCS patients into MCS+ and MCS−.

**Table 1:** Demographics and clinical data of the included patients.

DOC	Age	DSI	Etiology	CRS-R				Recovery
				T0	T1	T2	T3	
MCS (*n* = 20)	40–45	270	Hemorrhage	MCS−	MCS−	MCS−	MCS−	N
	50–55	30	Hemorrhage	MCS−	MCS−	MCS−	MCS+	Y
	30–35	180	Hemorrhage	MCS−	MCS−	MCS−	MCS+	N
	26–30	270	TBI	MCS−	MCS+	MCS+	MCS+	N
	50–55	60	TBI	MCS−	MCS−	MCS−	MCS−	Y
	36–40	30	Hemorrhage	MCS−	MCS−	MCS−	MCS+	Y
	70–75	120	Hemorrhage	MCS−	MCS−	MCS−	MCS−	N
	60–65	90	Hemorrhage	MCS−	MCS−	MCS−	MCS+	Y
	60–65	90	Hemorrhage	MCS−	MCS−	MCS−	MCS−	N
	66–70	60	TBI	MCS−	MCS−	MCS−	MCS+	Y
	66–70	60	Hemorrhage	MCS−	MCS−	MCS−	MCS+	Y
	50–55	30	TBI	MCS−	MCS−	MCS−	MCS+	Y
	50–55	30	Hemorrhage	MCS−	MCS−	MCS−	MCS−	Y
	36–40	320	Hemorrhage	MCS−	MCS−	MCS−	MCS−	Y
	56–60	60	Hemorrhage	MCS−	MCS−	MCS−	MCS−	N
	66–70	60	Hemorrhage	MCS−	MCS−	MCS+	MCS+	Y
	66–70	60	Hemorrhage	MCS−	MCS−	MCS−	MCS−	N
	66–70	30	Hemorrhage	MCS−	MCS−	MCS+	MCS+	Y
	66–70	60	Hemorrhage	MCS−	MCS−	MCS−	MCS−	N
	60–65	60	Hemorrhage	MCS−	MCS−	MCS+	MCS+	Y
UWS (*n* = 15)	36–40	180	Hemorrhage	UWS	UWS	UWS	UWS	N
	46–50	60	Hemorrhage	UWS	UWS	UWS	UWS	N
	60–65	90	Hemorrhage	UWS	UWS	MCS−	MCS−	N
	50–55	60	Hemorrhage	UWS	UWS	MCS−	MCS−	Y
	50–55	60	Hemorrhage	UWS	MCS−	MCS−	MCS+	Y
	50–55	180	TBI	UWS	UWS	UWS	UWS	N
	36–40	240	Hemorrhage	UWS	UWS	UWS	UWS	N
	40–45	90	Hemorrhage	UWS	UWS	UWS	UWS	N
	50–55	90	TBI	UWS	UWS	UWS	UWS	Y
	66–70	90	TBI	UWS	UWS	UWS	UWS	N
	46–50	60	TBI	UWS	MCS−	MCS−	MCS−	Y
	60–65	90	Hemorrhage	UWS	UWS	UWS	UWS	N
	60–65	120	Hemorrhage	UWS	MCS−	MCS−	MCS−	N
	40–45	30	Hemorrhage	UWS	UWS	UWS	UWS	N
	50–55	30	Hemorrhage	UWS	UWS	UWS	UWS	N

*DSI, days since injury; TBI, traumatic brain injury; Y, yes; N, no. Recovery: the patient’s consciousness has returned to the normal level*.

The patients were divided into UWS (three females and 12 males, 51.0 ± 9.6 years old) and MCS (10 females and 10 males, 52.3 ± 16.9 years old) groups according to their CRS-R scores before treatment. DOC patients who had previously undergone tDCS treatment within the past month were excluded from the present study. The patients with aneurysm clips, pacemakers, other implanted devices, or other drugs that may influence the patients’ EEGs were also excluded from this study. This study was approved by the ethics committee of the Zhengzhou Central Hospital Affiliated to Zhengzhou University. Assessments on whether any of the included patients had been returned to the normal level were completed in June 2019.

### Experimental and HD-tDCS

#### Experimental Process

**Figure 1:**
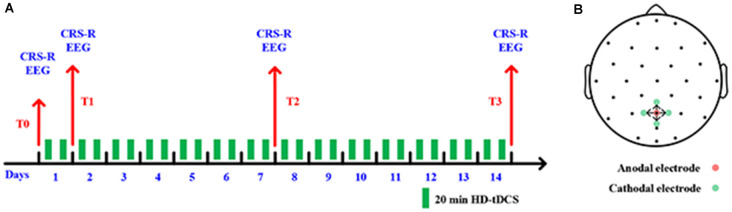
**(A)** Outline of the experimental design. **(B)** Positions of electrodes for high-definition transcranial direct-current stimulation (HD-tDCS).

As shown in Figure 1A, the experimental process consists of a consecutive 14 days HD-tDCS (position: anode centered over the precuneus; duration: 20 min; current: 2 mA) modulation. Every day each patient received two sessions stimulation in the morning and afternoon. We assessed the state of the patients’ consciousness with CRS-R at the following four time-points: the onset time of the experiment (T0), after 1 day of HD-tDCS (T1), after 7 days (T2), and the endpoint time of 14 days (T3). No adverse effect was found during the treatment.

#### HD-tDCS

In this study, five high-definition electrodes with an anode center electrode overlying the targeted brain area surrounded by four cathodal electrodes were used to transmit direct current to the brain (Model 4x1-C2: Soterix Medical Inc., New York, NY, USA). More restricted cortical neuromodulation and higher electric fields can be obtained by HD-tDCS (Villamar et al., 2013). Stimulating electrodes were arranged with specially designed plastic casings embedded in a 32-channel EEG recording cap. A previous study found that tDCS on the posterior parietal cortex could increase the CRS-R score for some DOC patients with MCS (Huang et al., 2017). As such, we placed the center anode at Pz position in line with the international 10-20 EEG system; the four cathodal electrodes were deployed approximately 3.5 cm radially from Pz position, approximately at Cz, P3, P4, and POz, as shown in Figure 1B.

### Data Recording and Pre-processing

A 32-channel EEG recorder was used for data collection (Nicolet EEG V32, Natus, United States; Channel names: Fp1, Fp2, F3, F4, C3, C4, P3, P4, O1, O2, F7, F8, T7, T8, P7, P8, A1, A2, Fz, Cz, Pz, FC5, FC1, FC2, FC6, POz, CP1, CP2, CP6, Fpz, CP5, and Oz). Resting-state EEGs were recorded at four time-points (T0, T1, T2, and T3). The 32 channels EEG signals were continuously recorded covering the whole scalp consistent with the International 10/20 system with the Cz as reference. The signals were notch filtered at 50 Hz in the recorder. The sampling rate of EEG signals was set to 1,000 Hz. During the data collection process, the impedance of all electrodes was kept no more than 5 KΩ and the patients were behaviorally awake. The EEG recording lasted approximately 10 min for each session.

All EEG data from the 32 channels were retained for analysis using EEGLAB (Delorme and Makeig, 2004). EEG signals were bandpass filtered at 0.5 and 45 Hz with the zero-phase shift FIR filter and then were segmented into 10 s epochs (approximately 60 epochs). Relative to the mean voltage of the entire epoch, every epoch was baseline-corrected at each time point. To select the abnormal channels, the normalized variance of each channel was calculated and then manually interpolated with the surrounding electrodes. We used independent component analysis (ICA) to reject other artifacts (EMG, EOG, and ECG; Jung et al., 2000). The artifacts were firstly selected with an automatic EEG artifact detector (Mognon et al., 2011). The EEG signals were re-referenced to the common average reference (CAR; Nunez et al., 2001). The first 40 clean epochs from each patient were preserved for further analysis.

### Construction of Brain Networks

The debiased weighted phase lag Index (dwPLI) was used for estimating spectral connectivity between pairs of channels. The dwPLI measuring of phase relationships is an estimator of scalp-level connectivity that is more robust and partially invariant to volume conduction in comparison to other estimators (Peraza et al., 2012). In the present study, the dwPLI measure was computed to estimate the functional connectivity between electrodes (Vinck et al., 2011) using the FieldTrip toolbox in MATLAB (Oostenveld et al., 2011).

First, we calculated cross-spectral decompositions from cleaned EEG datasets. Then, to achieve the dwPLI measure, the cross-spectrum between the spectral decompositions of every pair of channels was calculated. Furthermore, we restricted the analysis to the delta (1–4 Hz), theta (4–8 Hz), alpha (8–13 Hz), beta (13–30 Hz), and gamma (30–45 Hz) bands. Within each band, the dwPLI values across all epochs were averaged to represent the connectivity between channel pairs. For each patient’s dataset, the dwPLI values across all channel pairs were used to construct symmetric 32 × 32 × 4 dwPLI connectivity matrices for the delta, theta, alpha, beta, and gamma bands. Finally, each dwPLI matrix estimated above was proportionally thresholded. Values below this threshold were set to zero and other values were set to one, and 35% of the connectivity remained after thresholding. The threshold for each DOC patient was determined based on all four condition pools (T0, T1, T2, and T3) and was then applied to each condition separately. The binarized dwPLI connectivity matrices were used for further analysis. Due to the A1 and A2 electrodes being next to the face and vulnerable to noise, these two electrodes were excluded from the subsequent analysis. Finally, we obtained a symmetric 30 × 30 × 4 binarized dwPLI matrix for each band of each patient.

### Brain Network Measures

Two network measures, clustering coefficient (Watts and Strogatz, 1998) and global efficiency (Latora and Marchiori, 2001) were used to explore the properties that refer to the information processing in the human brain network. The brain-network measurement algorithms were implemented by the Brain Connectivity Toolbox (Rubinov and Sporns, 2010).

The clustering coefficient was computed as follows:

(1) $$clustcoef_{i}=\frac{2\sum_{j=1}^{n}v_{ij}}{n_{i}(n_{i}-1)}$$

in which *i*, *j* refers to the electrode number, *v*_ij_ is 1 if there is suprathreshold connectivity between electrodes *i* and *j* and 0 otherwise, and *n* is the number of electrodes with suprathreshold connections to electrode *i*. The clustering coefficient reflects the integrity and interconnectedness of a smaller network. Namely, it reflects local information processing. To get the average clustering coefficient of a network, the clustering coefficient values over all 30 channels were averaged for each patient.

Global network efficiency is closely related to the characteristic path length. The formula to compute global efficiency is as follows:

(2) $$E_{glob}=\frac{1}{N(N-1)}\sum_{i\neq j}\frac{1}{l_{ij}}$$

where *l*_ij_ indicates the shortest path length from the node *j* to the node *i*. Global network efficiency could reflect the efficiency of global information exchange in brain networks.

### Statistical Analysis

To test whether the clustering coefficient and global efficiency were increased at T1, T2, and T3 compared to T0 in both VS and MCS groups, the single tail paired *t*-test was used. Ten repeated statistical tests (2 network properties × 5 EEG bands) were performed at each time point for the two groups. To reduce the false-positive rate for multiple comparisons, statistical tests were corrected with the BHFDR method (Benjamini and Hochberg, 1995). The similar statistical analysis method and corrected methods were then used to test whether the nodal dwPLI values were increased at T1, T2, and T3 compared to T0, only the repeated times were different, which was 30 in this test. When testing the significant changes of CRS-R scores, the beta/gamma-band average clustering coefficient, and global network efficiency between the two groups, a two-sample *t*-test, and BHFDR correction methods were used and the repeated times is five.

## Results

Table 1 gives an account of the demographic and clinical data of the included patients. There are no significant differences between the UWS and MCS groups, except for CRS-Rs at the T0 phase. For the MCS group, there were 11 (55%) patients who showed improved consciousness states at the T3 phase and 12 (60%) patients had a recovery. Moreover, nine of 12 (75%) recovery patients had behaviorally improved. For the UWS group, there were five (33%) patients who showed improved consciousness states at the T3 phase and four (27%) patients had a recovery. Interestingly, three of four (75%) recovery patients had behaviorally improved.

### Changes in Resting-State Brain Network Properties

**Figure 2:**
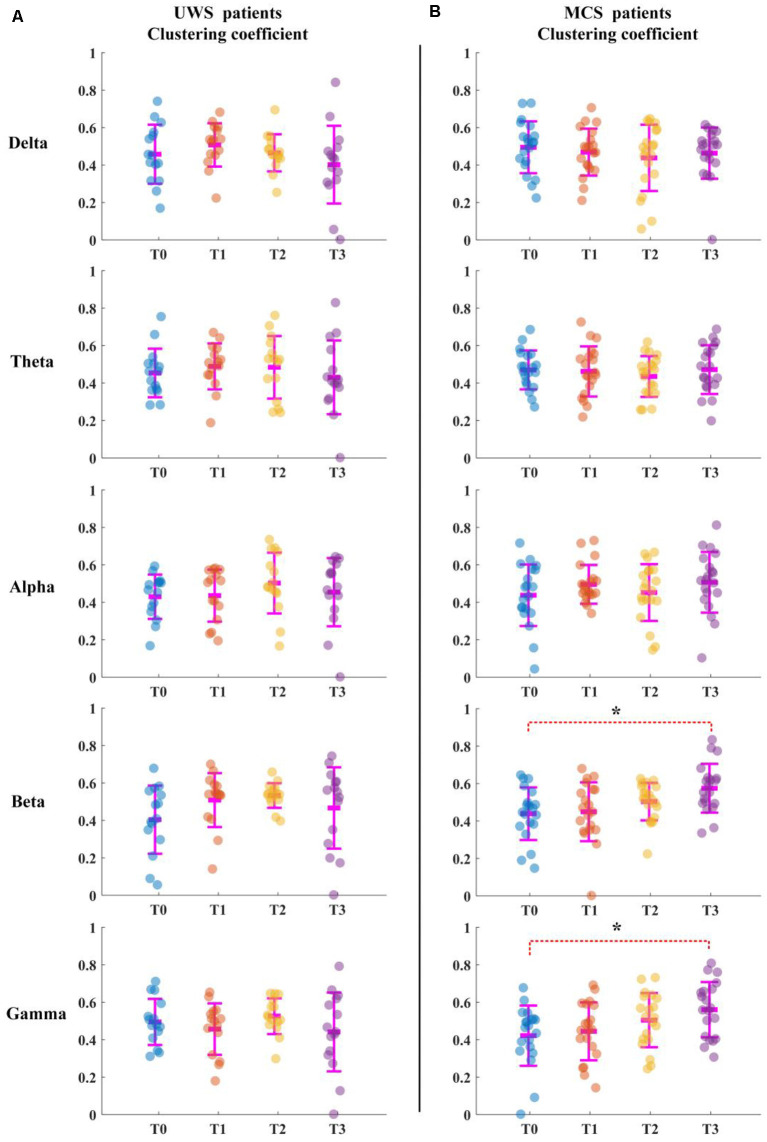
Average clustering coefficient for all patients in five bands. T0, T1, T2, and T3 represent four electroencephalograms (EEG) time points. The scatterplot shows the indicators of each patient. **(A)** Unresponsive wakefulness syndrome (UWS) patients. **(B)** Minimally conscious states (MCS) patients. *Denotes *p* < 0.05, FDR corrected.

**Figure 3:**
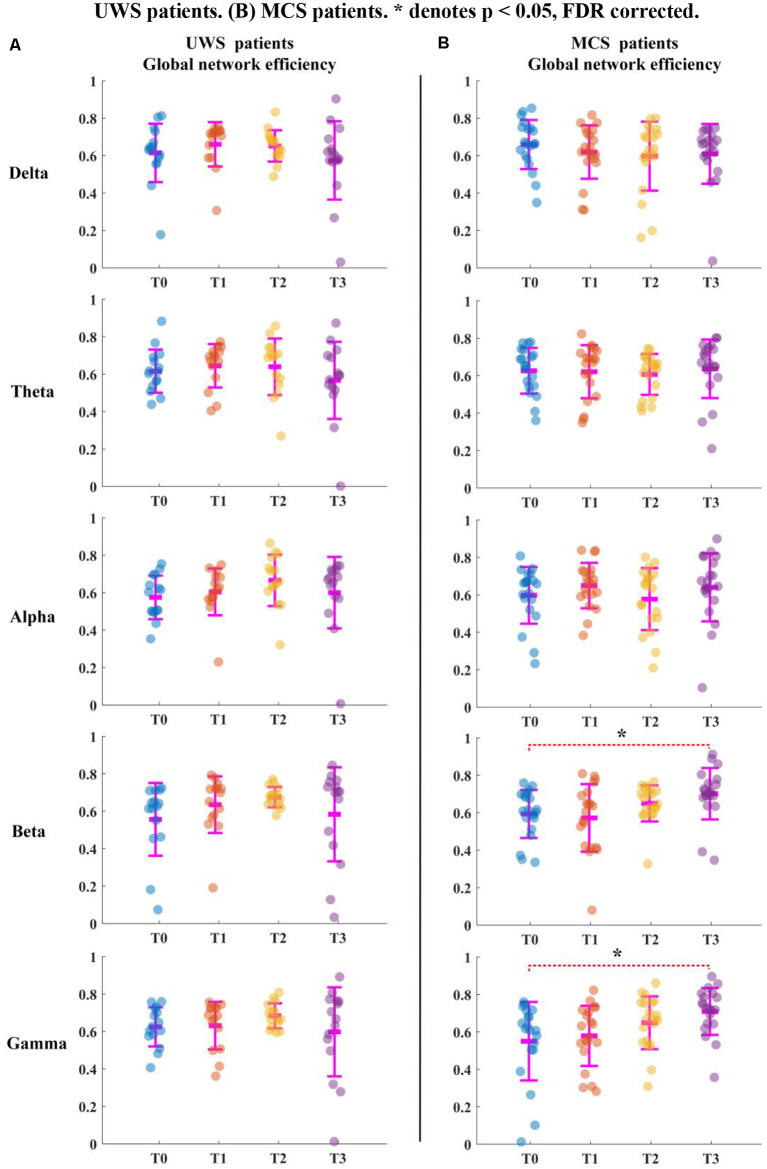
Global network efficiency for all patients in five bands. T0, T1, T2, and T3 represent four EEG time points. The scatterplot shows the indicators of each patient. **(A)** UWS patients. **(B)** MCS patients. *Denotes *p* < 0.05, FDR corrected.

For the UWS patients, as shown in Figure 2A, the average clustering coefficient values in the delta band decreased with increasing sessions of HD-tDCS; however, this trend was not significant (*p* > 0.05, FDR corrected). The average clustering coefficient values in theta, alpha, beta, and gamma bands did not show any significant change at the T3 phase compared with those of the T0 phase. The results in Figure 3A were the same as the above results.

For the MCS patients, as shown in Figure 2B, the average clustering coefficient values in beta (*p* < 0.05, FDR corrected) and gamma (*p* < 0.05, FDR corrected) bands increased with sessions of HD-tDCS. The average clustering coefficient values in the delta band decreased at the T3 phase; however, this trend was not significant (*p* > 0.05, FDR corrected). The average clustering coefficient values in alpha and beta bands did not show a significant change at the T3 phase compared with those at the T0 phase. As shown in Figure 3B, the global efficiency values in the delta band decreased (*p* > 0.05, FDR corrected). In the beta and gamma bands, the global efficiency values increased with increasing sessions of HD-tDCS (*p* < 0.05, FDR corrected). In the delta and alpha bands, the global efficiency values did not show a significant change.

**Figure 4:**
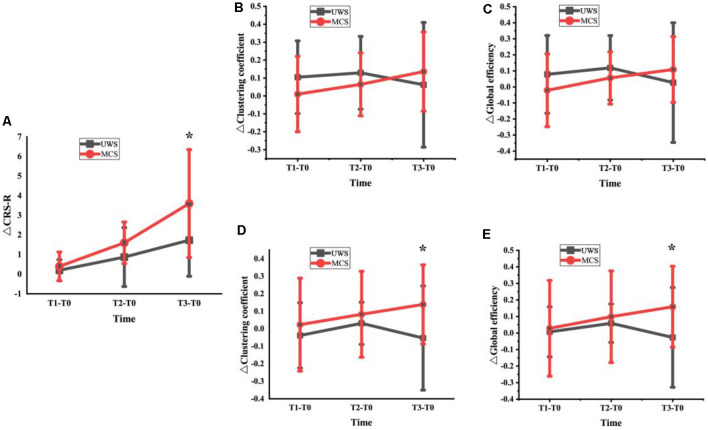
Changes of CRS-R scores **(A)**, average clustering coefficient and global network efficiency at T1, T2 and T3 for UWS and MCS groups [Beta band: **(B)** and **(C)**; Gamma band: **(D)** and **(E)**; *denotes a significant difference (*denotes *p* < 0.05, FDR corrected)].

We analyzed the changes in CRS-R scores, average clustering coefficient, and global network efficiency at T1, T2, and T3 for UWS and MCS groups. As showed in Figure 4A, although the two groups both showed a score increase, the MCS patients had a higher score increase. The increased CRS-R scores in the MCS group significantly higher than in the UWS group at the T3 phase (*p* < 0.05, FDR corrected). In Figures 4B,C, the beta band changes of the average clustering coefficient and global network efficiency increased in T1 and T2 phases but decreased in T3 for the UWS group. For the MCS group, although it didn’t show a significant difference at the T3 phase between the two groups, the changes increased steadily with the HD-tDCS treatment. In Figures 4D,E, the gamma band changes of average clustering coefficient and global network efficiency were similar to above beta band results, but it showed significant difference (*p* < 0.05, FDR corrected) at T3 between the two groups.

### Changes in Average Nodal Connection Strength

The average clustering coefficient and global efficiency values could not estimate which brain regions function increased or decreased after 14 days of HD-tDCS therapy. Therefore, the average nodal ΔdwPLI topographs of UWS and MCS patients in the five frequency bands were plotted. To obtain the average nodal ΔdwPLI of each node, the symmetric 30 × 30 × 4 binarized dwPLI matrix was averaged by rows; then, we obtained the average nodal connection-strength matrix (30 × 4). Finally, the nodal ΔdwPLI matrix (30 × 1) was obtained by calculating the difference between the four phases (T1-T0, T2-T0, and T3-T0).

**Figure 5:**
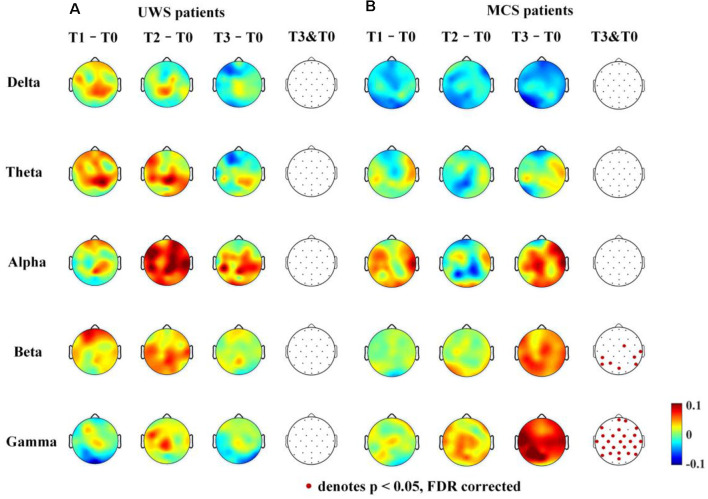
Average nodal ΔdwPLI (debiased weighted phase lag index) topographs (T1 – T0, T2 – T0 and T3 – T0) in UWS and MCS patients in terms of delta (1–4 Hz), theta (4–8 Hz), alpha (8–13 Hz), beta (13–30 Hz), and gamma (30–45 Hz) bands. The electrodes with significantly increased dwPLI (T0 →T3) are denoted by colored solid circles (**A**, UWS patients; **B**, MCS patients).

For the UWS patients, as shown in Figure 5A, there were no significantly increased nodal dwPLI values in the delta, theta, alpha, beta, or gamma bands between the T0 and T3 phase. Although the nodal dwPLI values increased in some regions in terms of theta, alpha, beta, and gamma bands for the T2 phase. But the results didn’t show statistical significance.

For the MCS patients, as shown in Figure 5B, the mean nodal dwPLI values decreased in the delta band. In the theta and alpha band, there were no significant changes in the mean nodal dwPLI values. In the beta band, the mean nodal dwPLI values increased in FC2, CP5, CP6, T8, P3, P7, P8, and POz (*p* < 0.05, FDR corrected). In the gamma band, the mean nodal dwPLI values increased in all electrodes (*p* < 0.05, FDR corrected), except Fp1, F3, F4, O1, O2, and Oz.

The changes in the nodal connection strength for MCS patients had a consistent increasing/decreasing trend during the HD-tDCS intervention. In contrast, for UWS patients, the changes in the node connection strength were not consistent. Additionally, the connection strength may have increased at the T2 phase, and then decreased at the T3 phase.

## Discussion

In this study, based on scalp EEGs, we probed the changes in resting-state functional networks for the UWS and MCS patients who received 14 days of HD-tDCS interventions. Additionally, we investigated whether there was any improved functional connectivity in different brain regions of these UWS and MCS patients. We found that local and global efficiency increased in beta and gamma bands after intervention for MCS patients. In contrast, UWS patients did not show any significant change in these two bands. Furthermore, for MCS patients, the increase of functional connectivity was distributed across nearly all brain regions, which is inconsistent with our experience that the sequence of functional rehabilitation of brain regions is in accordance with the distance of the stimulus area.

Studies had shown that anodal tDCS of the left DLPFC leads to cognitive improvement in healthy volunteers (Fregni et al., 2005; Ohn et al., 2008), hence, most studies selected the left DLPFC as stimulation target of DOC patients and verified that it is safe and can improve the state of consciousness for some chronic patients with MCS (Thibaut et al., 2017). While the precuneus was used in our study and the reasons are as follows. On the one hand, some studies’ results demonstrated that the precuneus may play a central role in the neural network correlates of consciousness (Laureys et al., 2006; Cavanna, 2007). On the other hand, the left DLPFC is common in traumatic brain injury, so an alternative stimulation target is needed for the patients with DLPFC injury. Our results proved that the stimulation on precuneus with HD-tDCS could benefit the recovery of MCS patients.

### Increased Network Local and Global Information Processing After HD-tDCS Intervention

In this study, we found that there was an increasing trend in local and global information processing of resting-state functional brain networks during the 14 days of HD-tDCS interventions. Although UWS patients did not show any significant change from the T0 phase to the T3 phase, we still observed that information processing was increased. The electrophysiological findings were consistent with clinical statistical results (Table 1). For the MCS group, there were more patients exhibited significant improvements at T3 compared with the UWS group, and most of the patients with improved consciousness states had behaviorally improved. This finding was consistent with conclusions from previous studies. A previous study showed that the brain networks of DOC patients had reduced local and global efficiencies compared with those of healthy controls (Chennu et al., 2014). The increased local and global efficiencies after 14 days of HD-tDCS interventions suggested that HD-tDCS may be useful for treating DOC patients. Our recent study also demonstrated that long-lasting HD-tDCS over the precuneus is promising for the treatment of DOC patients (Guo et al., 2019). Rizkallah et al. (2019) reported that resting-state brain networks of DOC patients showed significantly increased local information processing when compared to that of controls. Similar results were reported by Chennu et al. (2017), in which they found that there was a linear trend with an increasing level of consciousness in local information processing of alpha-band brain networks.

The reasons why HD-tDCS interventions had better effects on the MCS group compared to the UWS group need to be further investigated. One possible reason is that the MCS patients have more residual functional connectivity of the default mode network (DMN; Naro et al., 2017). Such functional connectivity is an indicator of covert consciousness, and the HD-tDCS may indirectly modulate other brain networks through the residual DMN.

Surprisingly, our findings mainly revealed significant differences *via* HD-tDCS interventions in terms of beta and gamma bands. The beta frequency range is related to selective attention, large-scale neuronal integration, states of the motor system, and environmental stimuli (Gilbertson et al., 2005; Engel and Fries, 2010; Donner and Siegel, 2011; Bonfiglio et al., 2014); gamma oscillatory activity is correlated with the level of awareness (Naro et al., 2016; Koch et al., 2016; Siclari et al., 2017). In our present study, the increased network of local and global information processing in beta and gamma bands suggests that there was the amelioration of symptoms in the included MCS patients.

### Increased Functional Connections Were Not Limited to the Stimulation Area

Another important finding was that the recovered functional connections in the beta and gamma bands were not only in the local brain area surrounding the stimulation position but were distributed across a more global brain area. This is not entirely inconsistent with our expected results; we originally hypothesized that the brain areas close to the stimulus position would be more likely to recover. Such results may be caused by the following two reasons. First, coma patients have been found to have a lower number of significant functional connections (Chennu et al., 2014; Malagurski et al., 2019), whereas MCS patients have been demonstrated to retain whole-brain functional connectivity (van den Heuvel et al., 2017; Malagurski et al., 2019). As such, stimulus information may be transmitted to other brain regions through these reserved connections. Additionally, although some specific brain regions or networks are specialized for performing specific tasks (He et al., 2018), integration of information cannot be done without whole-brain functional connectivity for complex cognition and behavior. Hence, the recovery of consciousness relies on strengthening of whole-brain functional connectivity.

### Limitations

Our study has two limitations. On the one hand, due to the patient’s family’s strong expectations for the recovery of consciousness, the sham stimulation group that lasted for 2 weeks did not recruit enough volunteers. So this study is not a random double-blind sham-controlled trial, thus the evidence obtained in the current study is not strong enough. For future HD-tDCS studies, adding a control group is necessary. On the other hand, although we have already recruited a lot of volunteers, the number is not big enough, more volunteers should be recruited for future study for making the results more convincing.

## Conclusion

Long-lasting HD-tDCS over the precuneus facilitated the recovery of consciousness for MCS patients. The increased clustering coefficient and global efficiency of resting-state brain networks within beta and gamma bands further supported the positive efficacy of HD-tDCS interventions. Additionally, we found that recovery of brain connectivity not only occurred in the local brain area surrounding the stimulation position but also occurred in more global brain areas. The results suggest that long-lasting HD-tDCS on the precuneus may facilitate information processing among neural populations in MCS patients.

## Data Availability Statement

The raw data supporting the conclusions of this article will be made available by the authors, without undue reservation.

## Ethics Statement

The studies involving human participants were reviewed and approved by The ethics committee of the Zhengzhou Central Hospital Affiliated to Zhengzhou University. The patients/participants provided their written informed consent to participate in this study. Written informed consent was obtained from the patient for the publication of any potentially identifiable data included in this article.

## Author Contributions

RZ: conceptualization, methodology, software, and writing—reviewing and editing. LZ: conceptualization, methodology, software, and writing—original draft preparation. YG: conceptualization, methodology, and data curation. LS: investigation, funding acquisition, and supervision. JG: investigation, supervision, and validation. XW: investigation and supervision. YH: conceptualization, project administration, funding acquisition, investigation, and supervision.

## Conflict of Interest

The authors declare that the research was conducted in the absence of any commercial or financial relationships that could be construed as a potential conflict of interest.
